# A New Indoor Positioning System Architecture Using GPS Signals

**DOI:** 10.3390/s150510074

**Published:** 2015-04-29

**Authors:** Rui Xu, Wu Chen, Ying Xu, Shengyue Ji

**Affiliations:** 1 The Hong Kong Polytechnic University, Hong Kong, China; E-Mails: lswuchen@polyu.edu.hk (W.C.); xu.xu@connect.polyu.hk (Y.X.); jidifferent@gmail.com (S.J.); 2 China University of Petroleum (Huadong), Qingdao 266000, China

**Keywords:** GNSS, indoor positioning, pseudolite

## Abstract

The pseudolite system is a good alternative for indoor positioning systems due to its large coverage area and accurate positioning solution. However, for common Global Positioning System (GPS) receivers, the pseudolite system requires some modifications of the user terminals. To solve the problem, this paper proposes a new pseudolite-based indoor positioning system architecture. The main idea is to receive real-world GPS signals, repeat each satellite signal and transmit those using indoor transmitting antennas. The transmitted GPS-like signal can be processed (signal acquisition and tracking, navigation data decoding) by the general receiver and thus no hardware-level modification on the receiver is required. In addition, all Tx can be synchronized with each other since one single clock is used in Rx/Tx. The proposed system is simulated using a software GPS receiver. The simulation results show the indoor positioning system is able to provide high accurate horizontal positioning in both static and dynamic situations.

## 1. Introduction

The widespread use of location-based services requires accurate user positioning information both outdoors and indoors. In the outdoor environment, Global Navigation Satellite System (GNSS) effectively provides accurate user position and is widely used in personal navigation devices (e.g., the smartphone). In indoor situation applications, however, it is difficult for GNSS to provide reliable positioning information due to the 20–30 dB additional signal attenuation and blocking caused by buildings and construction materials [1].

In indoor environments, different positioning methods have been proposed, such as the ultrasound [2,3], infrared [4], Bluetooth [5], Wi-Fi [6], image recognition [7], inertial navigation [8], and pseudolite (or pseudo-satellite) [9,10,11]. The ultrasound-based indoor positioning system can provide positioning solution with an accuracy of 1–3 cm [2,3]. However, some special equipment is required for this system. If the mobile device is used to receive the ultrasound signals, people will hear noises due to the non-ideal impulse response of speakers [12]. The infrared and Bluetooth methods have short coverage distance [4,5]. To cover the whole indoor area, a large number of signal sources are needed. The same requirement is also true for Wi-Fi systems in order to improve the positioning accuracy. Additionally, commonly based on the Received Signal Strength (RSS) and fingerprinting methods, Wi-Fi positioning systems have some shortcomings, such as large positioning errors due to the RSS offset between reference and user devices and long-duration fingerprinting updates. The image recognition method needs an *a priori* database of considerable image nodes to identify the location, commonly involving increased memory size and computing load [7]. For inertial navigation, there are two typical shortcomings: the positioning accuracy depends on the initial location and the positioning error increases with time [8]. Compared to these methods, the pseudolite system has relatively larger coverage area (up to 50 km) and more accurate positioning solution (carrier phase) with 4–5 transmitters [13]. If the pseudolite system uses specially designed GPS-like signals, a commercial GPS receiver can be used with minor modifications of the firmware and/or software [11].

There are three types of pseudolite architectures for indoor positioning system, named pseudolite, repeater and repealite [14]. The pseudolite represents the typical pseudolite architecture. Several pseudolites are located at the corners of the building to simulate the satellite constellation. Each pseudolite generates and transmits GPS-like signals. Commonly, the generated signals and real GPS signals have the same signal structure but differ in carrier frequency, PRN code or navigation data [13,15]. These differences require hardware-level modifications of the user receiver. In addition, pseudolites have some more drawbacks, such as clock synchronization issues and, multi-path and near-far effects [16]. To overcome one or more of these drawbacks, two variant architectures, the repeater and repealite, are proposed. The repeater simplifies the synchronization of the constellation and reduces near-far effects. It consists of an outdoor antenna to collect GPS signals and several switching modules to forward the GPS signals. In this process, the GPS signal is amplified and transmitted without any further treatment [17]. To identify the switching module, each module transmits signals over a definite period while others are turned off. The signal switching period received by the indoor terminal corresponds to the time difference of arrival (TDOA) between two successive repeaters. Then, more than four TDOA measurements are used to estimate the position [17]. The repealite is a combination of pseudolite and repeater. It is able to overcome the problems of synchronization and multipath effects. Like the repeater, the repealite transmits received GPS signals, but the transmission on each antenna is continuous and delayed by different periods, which is the measurement of the user receiver [18,19].

This paper proposes a new architecture for the pseudolite-repeater-based indoor positioning system. The main idea of the method is that each satellite’s signal is extracted from the received GPS signals (commonly including multiple satellites’ signal) and forwarded using one indoor transmitting antenna. This process is realized using a Receiver/Transmitter and includes collecting the GPS signal from the outdoor receiving antenna, demodulating the GPS signal in the receiver component, repeating each satellite signal in the transmitter component and then forwarding different satellite signals via indoor transmitting antennas. The measurement of the system is the difference of pseudoranges measured by the indoor terminal and outdoor receiver. The pseudorange difference corresponds to the distance between the indoor terminal and transmitting antenna, and a signal delay bias. Four such measurements are then used in the position calculation. The advantages of the system include simple clock synchronization and no/minor modification of the commercial GPS receiver.

The system is described in Section 2. The positioning algorithm in the user terminal is introduced in Section 3. The simulation method and results are shown in Section 4. Conclusions of the paper are presented in Section 5.

## 2. System Structure

**Figure 1:**
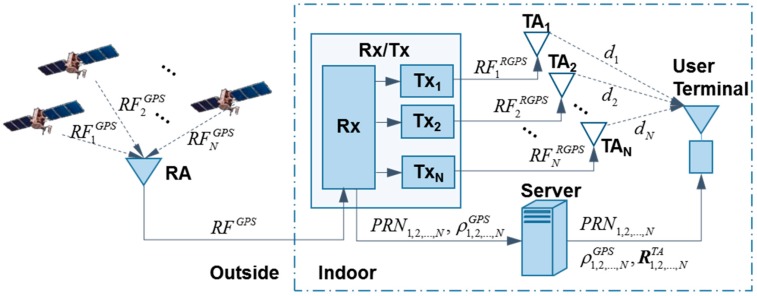
Indoor positioning system.

The proposed system is presented in Figure 1. It comprises a Receiver-and-Transmitter (Rx/Tx), server and user terminal.

### 2.1. Rx/Tx

**Figure 2:**
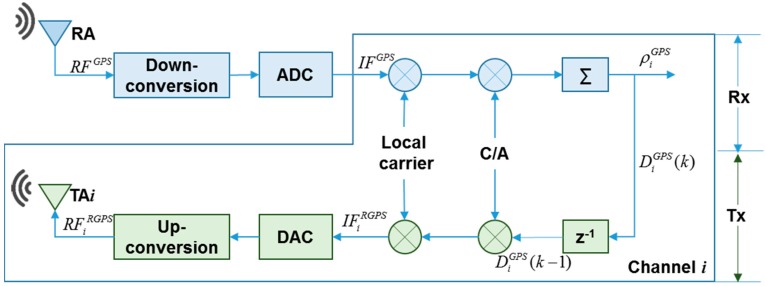
Architecture of Rx/Tx Server in one channel.

The Rx/Tx is the crucial part in the system. It receives the real GPS Radio Frequency (*RF^GPS^*) signal using an outdoor Receiving Antenna (RA), estimates the pseudoranges $\rho_{1,2,...,N}^{GPS}$ from the RA to each satellite and sends them to the server. Meanwhile, the Rx/Tx separates each satellite’s signal and transmits the separated signal to the user terminal via several (at least three for 2-D positioning) indoor Transmitting Antennas (TA_1_, TA_2_,…,TA_N_). The architecture of the Rx/Tx, which is composed of a receiver (Rx) and transmitter (Tx) components, is illustrated in Figure 2. The Rx component works as a general GPS receiver. Differently, its local carrier, code and navigation data of the receiver rather than positioning results are output to the next component (Tx).

The requirement, nowadays, can be satisfied by a software GNSS receiver. Figure 2 illustrates the signal processing in the Rx component. GPS radio frequency signals *RF^GPS^* is received by the outdoor RA, and converted into intermediate frequency signals *IF^GPS^* through down-conversion and Analog-Digital-Conversion (ADC). Since *IF^GPS^* is a mixture of multiple satellites’ signals, the Rx demodulates *IF^GPS^* using multiple channels and each channel processes one satellite’s signal. Through multiplying *IF^GPS^* by the local carrier and C/A code, the Rx obtains navigation data and estimates the pseudorange measurement $\rho_{i}^{GPS}$ from each channel. It sends the local carrier, local C/A code and demodulated navigation data to the Tx component. Simultaneously, it delivers all the pseudorange measurements $\rho_{1,2,...,N}^{GPS}$, their corresponding PRN and channel number to the server via Wi-Fi (or another possible means).

The Tx components mimic GPS satellites to generate and transmit RF. The navigation data, carrier and code signal are obtained from the signal processing channel of Rx. Thus, one Tx generates the GPS-like signal corresponding to one GPS satellite and one Rx channel. Figure 2 shows the process of the *i*-th Tx generating the RF signal. Firstly, Tx re-modulates IF signal using the local carrier, local C/A code and demodulated navigation data from the *i*-th channel of Rx. After re-modulation, the Tx*_i_* obtains the digital intermediate frequency signal $IF_{i}^{RGPS}$. Then, the signal $IF_{i}^{RGPS}$ is converted from digital into analog signal using Digital-Analog-Conversion (DAC) and further from IF signals into RF signal $RF_{i}^{RGPS}$ with L1 frequency (1575.42 MHz) using up-conversion. Finally, $RF_{i}^{RGPS}$ is sent to user terminal via TA*_i_*.

In the system, more than four Tx are required for 3-D positioning and more than three Tx are required for 2-D positioning since one Tx corresponds to one satellite. Therefore, synchronization of all Tx units is required in the system. In Figure 2, the time of RF signal generation and the time of signal transmission from Tx to TA are the main factors causing asynchronization, also called as system delay biases. For the signal generation time, Rx and all Tx are controlled using one oscillator to ensure that all Tx units generate RF signals simultaneously. The remained system delay biases can be calibrated in the following two ways:

(1) Pre-correction before installation of transmitting antennas

In this method, all transmitting antennas are placed at the same position and their transmitting signals are received using one GPS receiver. Then, the difference between the indoor receiver’s psuedorange measurements and the Rx component for the same satellite (*i.e.*, $\rho_{i}^{RGPS}-\rho_{i}^{GPS}$, denoted as $\Delta \rho_{i}$, which models will be detailed in Section 3, results from the processing time in Tx/Rx and propagation time from the transmitting antenna to the indoor receiver. The propagation time is the same since all transmitting antennas are at the same location. Therefore, in an ideal case of no delay in any channel, $\Delta \rho_{i}$ are the same in all channels and $\Delta \rho_{1}=\Delta \rho_{2}=...=\Delta \rho_{N}$ can be obtained. In practice, we likely obtain that $\Delta \rho_{1}\neq \Delta \rho_{2}\neq ...\neq \Delta \rho_{N}$ due to the system delay biases. Thus, the correction of system delay bias for each channel can be estimated using the difference of $\Delta \rho_{i}$. For instance, the correction of the *i*-th channel is $\Delta \rho_{i}-\Delta \rho_{1}$ without loss of generality. It should be noticed that this method estimates the different portions of system delay biases, but the common portions are cancelled by subtraction processing. The common portion will be included in the user clock error and estimated in positioning estimation in the user terminal.

(2) On-line correction using a reference indoor GPS station

As discussed in the pre-correlation, $\Delta \rho_{i}$ includes the system delay bias and the distance $d_{i}$ from the transmitting antenna to the indoor receiver. Herein, the correction of system delay bias can be estimated as $\Delta \rho_{i}-d_{i}$. $\Delta \rho_{i}$ can be estimated from the pseudorange measurements of indoor receiver and Tx/Rx. If the indoor receiver’s location can be obtained, $d_{i}$ will be known. Thus, in the on-line correction algorithm, a reference GPS station must be placed at a known location. Its distance to each antenna is a known constant and can be pre-estimated correctly. Using $\Delta \rho_{i}-d_{i}$, the correlation of system delay bias of each channel can be calculated. The correlation value can be deliver to user terminal via Wi-Fi.

In Figure 2, the demodulated navigation data $D_{i}(k)$ from the Rx is delayed by one epoch in Tx, where one epoch equals to one integration time $T_{coh}$ of tracking loop in the Rx. The reason for the one-epoch delay is that the navigation data bit can only be obtained after demodulation. In other words, the current navigation data bit cannot be obtained in the real-time system. Thus, the previous moment navigation data bit is used to reconstruct the RF signal and the minimum time delay is 1 ms ($T_{coh}$ = 1 ms). Since the delay occurs in every Tx, it will be considered as a part of receiver clock error and does not affect the positioning result.

### 2.2. Server

**Figure 3:**
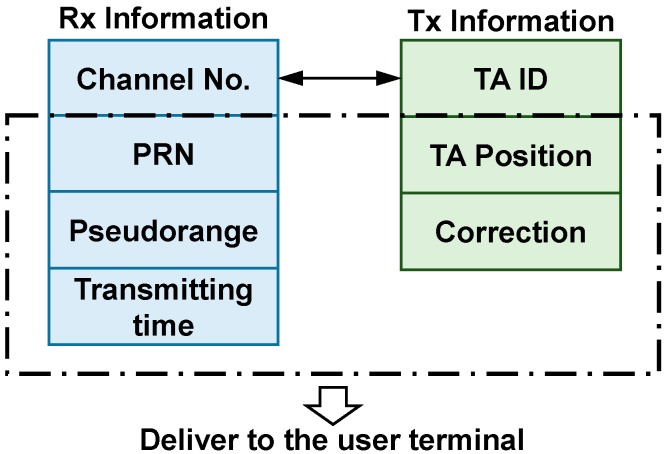
Rx and Tx information and information matching.

In the proposed system, the server is used to match the Rx and Tx information and deliver it to the user terminals via Wi-Fi. The Rx information includes the pseudorange measurements, corresponding transmitting time, PRN and channel number. This information is collected from Rx and updated on-line since pseudorange measurements and available satellites vary with time. The other reason for on-line updating is that the one-to-one relationship of channel number and its corresponding PRN is dynamically adjustable rather than fixed. The Tx information includes the position of each antenna which are required for position calculation. It also includes the identifier of transmitting antenna which relates to channel number. Using the identifier, the server can match the transmitting antenna for pseudorange measurement and PRN as shown in Figure 3. In addition, the transmitting antenna is likely to be far away from the receiving antenna due to a reasonable antenna distribution. As a result, the signal propagation time in the cable connecting transmitting antenna cannot be ignored. Therefore, the stored information also contains the delay correction of each channel referring to the signal propagation time delay.

### 2.3. User Terminal

**Figure 4:**
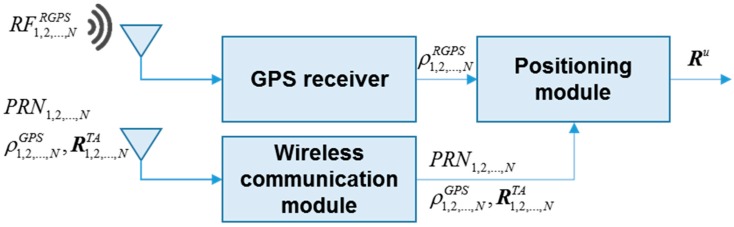
Architecture of the user terminal.

The user terminal includes a GPS receiver module, wireless communication module (*i.e.*, Wi-Fi) and positioning calculation module, as shown in Figure 4. The GPS receiver is used to track the mimicked GPS signals from the different transmitting antennas. It is also used to estimate the pseudorange measurements rather than user position. This is because the position estimated by the GPS receiver is not the real user position. The range from the user terminal to TA, which is required in positioning estimation, is contained in the pseudorange measurement from the GPS receiver module. The wireless communication module is used to acquire the Rx and Tx information from the server, which are also used to estimate the range to TA. The positioning module receives the pseudorange measurements from GPS receiver module and wireless communication module, calculating the range to each transmitting antenna, and then estimating the user position.

## 3. Measurement Model and Positioning Algorithm

As introduced in the previous section, the user position estimation depends on the range between user terminal and TAs, which is included in the pseudorange measured by the GPS receiver module of user terminal. The pseudorange measurements $\rho_{i}^{RGPS}$ from the user terminal to the *i*-th TA is modelled as:

(1) $$\rho_{i}^{RGPS}=\rho_{i}^{GPS*}+{\text{δ}}_{i}^{RT}+d_{i}+{\text{δ}}_{clc}^{u}+{\text{ε}}_{i}^{u}$$

where $\rho_{i}^{GPS*}$ represents the range due to real GPS signal reproduction in *i*-th Rx/Tx channel, ${\text{δ}}_{i}^{RT}$ represents the distance between the receiving and transmitting antenna, $d_{i}$ represents the range between the user terminal and the *i*-th TA, ${\text{δ}}_{clc}^{u}$ represents the range due to the clock error of user terminal and $\varepsilon_{i}^{u}$ represents the delay due to the thermal noise of the GPS module in the user terminal.

In the above equation, the term of $\rho_{i}^{GPS*}$ has the same description as $\rho_{i}^{GPS}$ since $\rho_{i}^{GPS*}$ is from the duplication of corresponding GPS signal, which is modelled as [20]:

(2) $$\rho_{i}^{GPS*}=\left|R^{RA}-R_{i}^{SAT}\right|+{\text{δ}}_{clk}^{RA}+{\text{δ}}_{clk,i}^{SAT}+{\text{δ}}_{ion,i}+{\text{δ}}_{trop,i}+{\text{δ}}_{mul,i}+{\text{ε}}_{i}^{Rx}$$

where $R^{RA}$ represents the position vector of receiving antenna, $R_{i}^{SAT}$ represents the position vector of *i*-th GPS satellite, ${\text{δ}}_{clk}^{RA}$ represents the clock error of Rx, ${\text{δ}}_{clk,i}^{SAT}$ represents the clock error of the *i*-th GPS satellite, ${\text{δ}}_{ion,i}$ represents the ionospheric range delay, ${\text{δ}}_{trop,i}$ represents the tropospheric delay, ${\text{δ}}_{mul,i}$ represents the multipath error, and $\varepsilon_{i}^{Rx}$ represents thermal noise of Rx.

If the user terminal synchronizes the time of $\rho_{i}^{GPS}$ and $\rho_{i}^{GPS*}$, $\rho_{i}^{GPS*}\approx \rho_{i}^{GPS}$ can be obtained. The synchronization is satisfied using satellite transmission time which is obtained from the C/A code on the real-world GPS signal and repeated GPS signal. Therefore, the difference between $\rho_{i}^{RGPS}$ and $\rho_{i}^{GPS}$ can be written as:

(3) $$\begin{array}{ll} \Delta {\text{ρ}}_{i} & ={\text{ρ}}_{i}^{RGPS}-{\text{ρ}}_{i}^{GPS}\\ & ={\text{ρ}}_{i}^{GPS*}+{\text{δ}}_{i}^{RT}+d_{i}+{\text{δ}}_{clc}^{u}+{\text{ε}}_{i}^{u}-{\text{ρ}}_{i}^{GPS}\\ & \approx {\text{δ}}_{i}^{RT}+d_{i}+{\text{δ}}_{clc}^{u}+{\text{ε}}_{i}^{u} \end{array}$$

The difference between $\rho_{i}^{RGPS}$ and $\rho_{i}^{GPS}$ removes the GPS signal propagation errors, such as ionospheric delay, tropospheric delay and orbit errors. As a result, the proposed indoor positioning system has high robustness to the effects of the GPS signal propagation environment. In addition, the difference cancels the errors due to the Rx component, such as the clock error. This indicates that the accuracy of the proposed system is independent of the accuracy of Rx and hence a low-cost GPS receiver can be used as the Rx component. The term $\delta_{i}^{RT}$ is a known quantity, saved in the server and delivered to the user terminal:

(4) $$\begin{array}{ll} \Delta {\text{ρ}}_{i} & =d_{i}+{\text{δ}}_{clc}^{u}+{\text{ε}}_{i}^{u}\\ & =\left\|R^{u}-R_{i}^{TA}\right\|+{\text{δ}}_{clc}^{u}+{\text{ε}}_{i}^{u} \end{array}$$

where $R^{u}={\left[\begin{array}{ccc} x^{u} & y^{u} & z^{u} \end{array}\right]}^{T}$ represents the unknown user position and $R_{i}^{TA}={\left[\begin{array}{ccc} x_{i}^{TA} & y_{i}^{TA} & z_{i}^{TA} \end{array}\right]}^{T}$ represents the position of *i*-th TA obtained from the server. Similar to GPS positioning algorithm, user position is estimated through solving the equation:

(5) $$(x^{u},\;y^{u},z^{u},{\text{δ}}_{clc}^{u})=\underset{\left(x^{u},\;y^{u},z^{u},{\text{δ}}_{clc}^{u}\right)}{\arg\;\min}\;\sum_{i=1}^{N}{\left(\left\|R^{u}-R_{i}^{TA}\right\|-\Delta {\text{ρ}}_{i}\right)}^{2}$$

Commonly, Equation (5) is solved using the least squares method.

## 4. Simulation and Results

**Figure 5:**
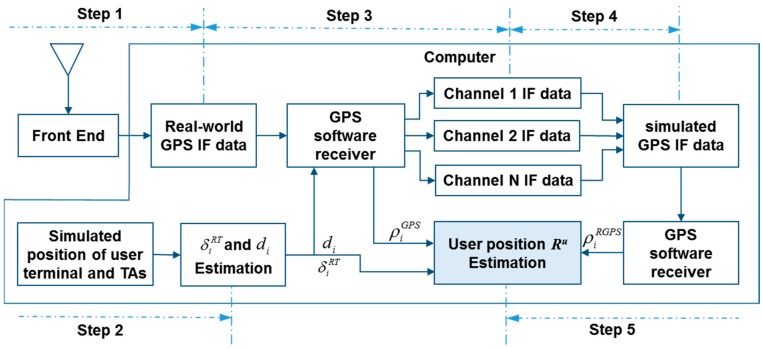
Simulation process.

In the simulation test, we used a software GPS receiver to realize the functions of Tx/Rx. The simulation test is performed in the following steps and illustrated in Figure 5:

*Step 1*: Collecting real-world GPS data. The real-world GPS signal is collected by a front end (iP-solutions GPS/Galileo L1 RF recorder with OCXO) and logged as a data file in the computer.

*Step 2*: Simulating TA distribution and user position, ${\text{δ}}_{i}^{RT}$ and $d_{i}$. The distance ${\text{δ}}_{i}^{RT}$ is estimated according to the simulated distribution of TAs and the range $d_{i}$ is estimated according to the simulated user position and TA position.

*Step 3*: Separating GPS IF data. The software GPS receiver is used to demodulate the real-world GPS data. The navigation data, carrier and code with delay due to ${\text{δ}}_{i}^{RT}$ and $d_{i}$ of each channel corresponding to one satellite are remodulated. The remodulated signal is used to simulate the indoor signal transmitted by the TA.

*Step 4*: Mixing the separated GPS IF data of different channels (*i.e.*, different satellites). The GPS IF data of different channels are combined into one IF data stream to simulate the GPS data collected indoors using the user terminal.

*Step 5*: Simulating the user terminal’s function. A software GPS receiver is used as the GPS module in the user terminal to process the simulated GPS IF data and output pseudorange measurements $\rho_{i}^{RGPS}$. The TA distribution, ${\text{δ}}_{i}^{RT}$ and $d_{i}$ are obtained from Step 2, the pseudorange measurements of outside GPS signal $\rho_{i}^{GPS}$ are obtained in Step 3. The user position is estimated according to Equation (5).

The parameters of the Front End and the GPS software receiver used in the simulation are shown in Table 1.

**Table 1:** Parameters of front end and GPS software receiver.

GPS Signal	L1
Sampling frequency	16.3676 MHz
Intermediate frequency	4.1043 MHz
Integration time	1 ms
PLL bandwidth	10 Hz
DLL bandwidth	1 Hz

**Figure 6:**
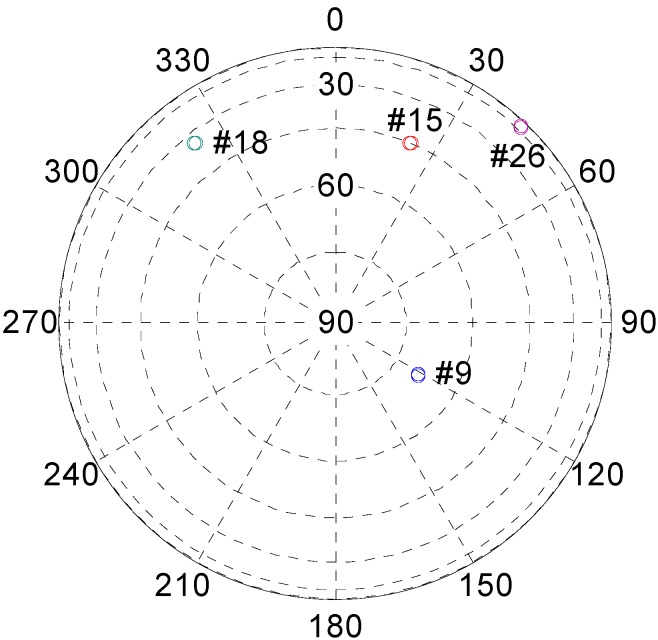
Sky plot.

In the simulation, a real-world GPS signal was collected on the roof of the Lui Che Woo building at the Hong Kong Polytechnic University in October, 2012. Four satellites’ signal are randomly selected to generate $RF_{i}^{RGPS}$. Their distributions are shown in Figure 6. The figure shows that the four satellites (PRN 9, 18, 15 and 26) have bad geometry. All of them are distributed in the section with 322.3°–121.8° azimuth angle. In addition, one of them (PRN 26) has very low elevation angle reaching 11.7°. As a result, the PDOP value reaches 4.43.

### 4.1. Static Simulation

**Figure 7:**
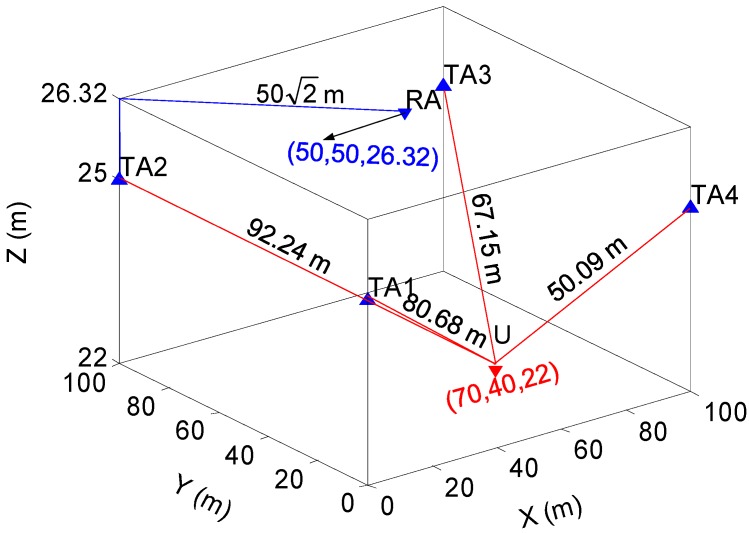
Trace of static test.

To test the effectiveness of proposed indoor positioning system, a static test is performed first. The distribution of the RA and four TAs is illustrated in Figure 7. RA is at the centre of the four TAs and at a height of 26.32 m, 1.32-m higher than the TAs’ heights since it is outside. The four TAs are on the same plane and located on the vertexes of a 100 m × 100 m square. Assuming the RA and each TA are connected using cables, the route between the TAs and the RA is $1.32+50\sqrt{2}\;\text{m}\approx 72.03\;\text{m}$. The user terminal is on the lowest layer and its real position in the local coordinates is (70, 40, 22).

**Figure 8:**
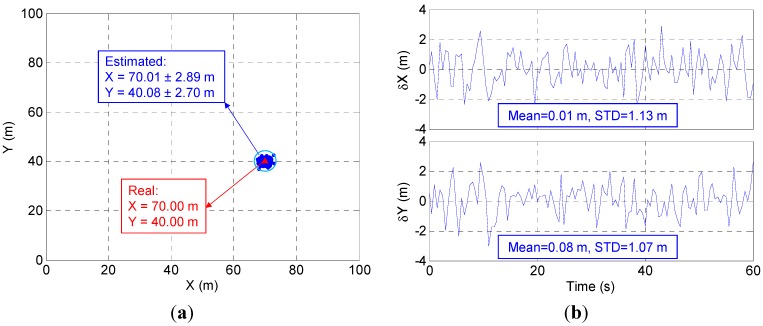
Static test results: (**a**) positioning results and (**b**) positioning error.

Figure 8 displays the 2-D positioning results of the static test with a fixed vertical value of 22 m. The results show that the proposed system is able to provide an indoor positioning solution as accurate as ~1 m. The average positioning error is close to zero, as small as 0.01 m in X axis and 0.08 m in Y axis. The maximum error is 2.89 m in the X axis and 2.70 m in the Y axis. The standard deviation (STD) of the X-axis error is 1.13 m and of the Y-axis error is 1.07 m. The results also suggest that the accuracy of the proposed system is not affected by the bad satellite geometry.

**Figure 9:**
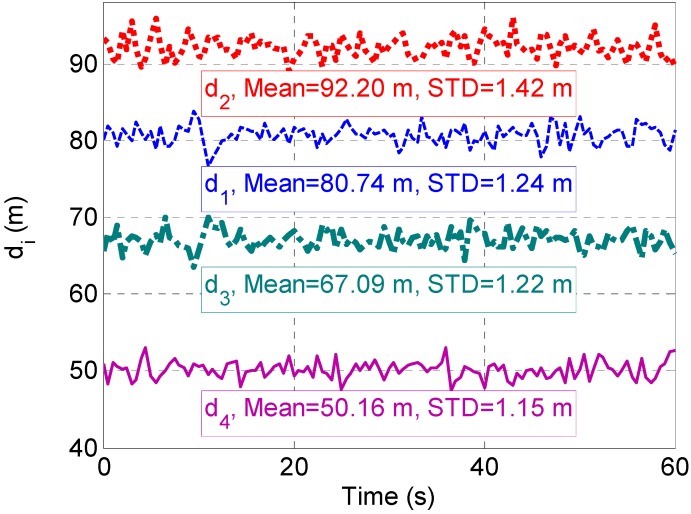
Estimated distance between the user terminal and each TA.

Additionally, the high accuracy of the proposed system results from the fact that the propagation errors, such as ionospheric delay, tropospheric delay and orbit error, are cancelled in calculating $\Delta \rho_{i}$ through $\rho_{i}^{RGPS}$ minus $\rho_{i}^{GPS}$ which has been shown in Equation (3). Thus, the distance from the user terminal to each TA can be accurately estimated according to Equation (5). Figure 9 shows that the estimated distances are similar to the real value. For instance, the real value of $d_{2}$ is 92.24 m. Its estimated result is 92.20 m with 1.42 m STD.

**Figure 10:**
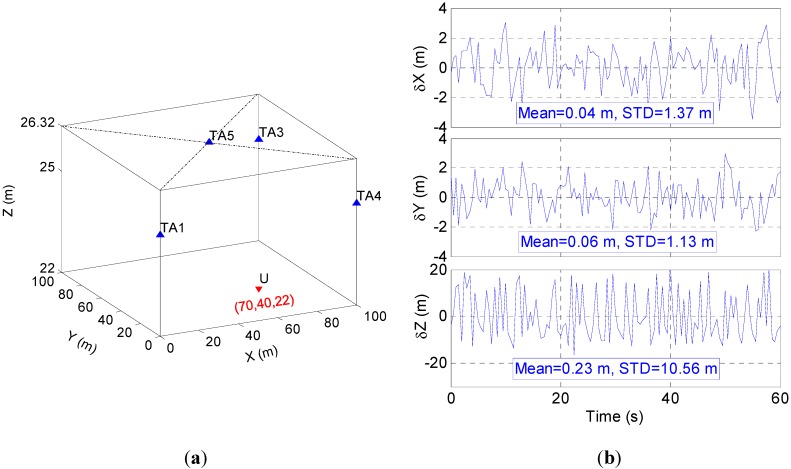
Distribution of TA for 3-D positioning test and its positioning error: (**a**) TA distribution; and (**b**) positioning error.

Figure 10 shows the antenna distribution for the 3-D positioning test with four transmitting antennas (a) and its positioning errors in three axes (b). In the figure, the positioning error is 0.04 m with 1.37 m STD in the X axis and 0.06 m with 1.13 m STD in the Y axis, which is similar to the 2-D positioning accuracy. Compared to the horizontal error, the Z-axis error is much larger, reaching 0.23 m with STD = 10.56 m. It should be noticed that the low accuracy in the Z-axis has little effect on the horizontal accuracy, as mentioned above. Additionally, the average error in the Z axis is 0.23 m which is acceptable to detect floors, although the Z-axis error changes in a large range.

### 4.2. Dynamic Simulation

**Figure 11:**
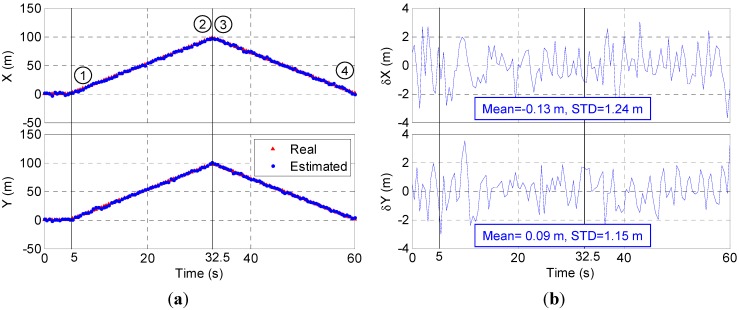
Dynamic simulation results: (**a**) positioning results and (**b**) positioning error.

The trace of dynamic simulation is from (1, 1, 22) to (99, 99, 22) and back to (1, 1, 22) with 5 m/s velocity as shown in Figure 11a (red triangle dots). The dynamic simulation results, displayed in Figure 11, show the proposed system also has high dynamic positioning accuracy although the error is a little larger than that under static conditions. The average error is −0.13 m in the X axis and 0.09 m in the Y axis. The STD is 1.24 m in the X axis and 1.15 m in the Y axis.

**Figure 12:**
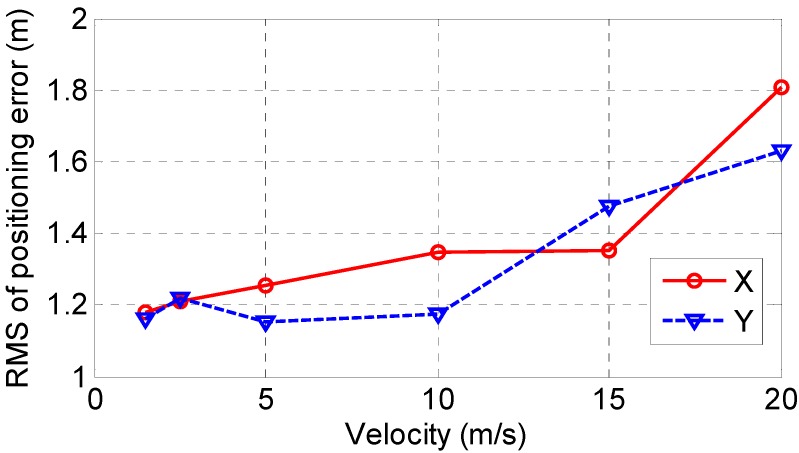
Positioning error variation with velocity.

More dynamic tests are conducted using back-and-forth trace between (1, 1, 22) and (99, 99, 22) with different velocities of 1.5 m/s (normal human walking speed), 2.5 m/s (jogging speed), 10 m/s, 15 m/s and 20 m/s. The Root Mean Square (RMS) of the X-axis and Y-axis errors are summarized in Figure 12. The simulation results show the positioning accuracy in the X-axis and Y-axis generally degrades with increasing velocity. In the case of velocity = 20 m/s, the X-axis error is 1.81 m, 0.56 m larger than that in the case of velocity = 5 m/s. The Y-axis error increases from 1.15 m at velocity = 5 m/s to 1.63 m at velocity = 20 m/s. The increase reaches 0.56 m. As the human normal walking and jogging speeds are less than 5 m/s, the proposed system is able to provide position estimation with accuracy of about 1.2 m in the X-axis and Y-axis for persons.

## 5. Conclusions

This paper proposes a new pseudolite-based indoor positioning system. The proposed system includes a Receiver-and-Transmitter (Rx/Tx), server and user terminal. As the kernel of the system, Rx/Tx demodulates the received real-world GPS signal (which includes several satellites’ signals), separates them and repeats them, respectively. This process has two advantages. One is that all Tx can be synchronized with each other since one single clock is used in the Rx/Tx system. The other advantage is the GPS-like signal can be processed (signal acquisition and tracking, navigation data decoding) by the general receiver and hence there is no need for hardware-level modification on the receiver. The proposed system is simulated using a software defined GPS receiver. The simulation results show the indoor positioning system is able to provide high accurate horizontal positioning in both static and dynamic situations. In the future, the study will focus on the realization problem, such as indoor transmitting antennas distribution, multi-path effects and so on.

## References

[1] Mautz R. (2009). Overview of current indoor positioning systems. Geod. Cartogr..

[2] Medina C., Segura J.C., de la Torre Á. (2013). Ultrasound indoor positioning system based on a low-power wireless sensor network providing sub-centimeter accuracy. Sensors.

[3] Ruiz D., Garcia E., Urena J., de Diego D., Gualda D., Garcia J.C. Extensive ultrasonic local positioning system for navigating with mobile robots. Proceedings of the 2013 10th Workshop on Positioning, Navigation and Communication.

[4] Oh J.H., Kim D., Lee B.H. (2014). An indoor localization system for mobile robots using an active infrared positioning sensor. J. Ind. Intell. Inf..

[5] Lee S., Koo B., Jin M., Park C., Lee M.J., Kim S. Range-free indoor positioning system using smartphone with bluetooth capability. Proceedings of the 2014 IEEE/ION Position, Location and Navigation Symposium.

[6] Liu H.-H., Lo W.-H., Tseng C.-C., Shin H.-Y. (2014). A wifi-based weighted screening method for indoor positioning systems. Wirel. Pers. Commun..

[7] Liang J.Z., Corso N., Turner E., Zakhor A. Image based localization in indoor environments. Proceedings of the 2013 Fourth International Conference on Computing for Geospatial Research and Application.

[8] Collin J., Mezentsev O., Lachapelle G. Indoor positioining system using accelerometry and high accuracy heading sensors. Proceedings of the ION GPS/GNSS 2003.

[9] Puengnim A., Patino-Studencka L., Thielecke J., Rohmer G. Precise positioning for virtually synchronized pseudolite system. Proceedings of the 2013 International Conference on Indoor Positioning and Indoor Navigation.

[10] Selmi I., Vervisch-Picois A., Gottesman Y., Samama N. GNSS-based calibration of the infrastructure of the repealite indoor positioning system. Proceedings of the 2013 International Conference on Indoor Positioning and Indoor Navigation.

[11] Kim C., So H., Lee T., Kee C. (2014). A pseudolite-based positioning system for legacy gnss receivers. Sensors.

[12] Lazik P., Rowe A. Indoor pseudo-ranging of mobile devices using ultrasonic chirps. Proceedings of the 10th ACM Conference on Embedded Network Sensor Systems.

[13] Rizos C., Roberts G., Barnes J., Gambale N. Experimental results of locata: A high accuracy indoor positioning system. Proceedings of the 2010 International Conference on Indoor Positioning and Indoor Navigation.

[14] Samama N., Jin S. (2012). Indoor positioning with gnss-like local signal transmitters. Global Navigation Satellite Systems—Signal, Theory and Applications.

[15] Rapinski J., Cellmer S., Rzepecka Z. (2012). Modified gps/pseudolite navigation message. J. Navig..

[16] Rapinski J., Koziar M., Rzepecka Z., Cellmer S., Chrzanowski A. (2012). Some considerations in designing a gps pseudolite. Artif. Satell..

[17] Fluerasu A., Jardak N., Vervisch-Picois A., Samama N. In GNSS repeater based approach for indoor positioning: Current status. Proceedings of the European Navigation Conference, Global Navigation Satellite Systems.

[18] Vervisch-Picois A., Samama N. First experimental performances of the repealite based indoor positioning system. Proceedings of the 2012 International Symposium on Wireless Communication Systems.

[19] Selmi I., Samama N., Vervisch-Picois A. A new approach for decimeter accurate GNSS indoor positioning using carrier phase measurements. Proceedings of the 2013 International Conference on Indoor Positioning and Indoor Navigation.

[20] Xu G. (2007). Theory, Algorithms and Applications.

